# Injuries in Cricket: A Review

**DOI:** 10.1007/s12178-026-10029-8

**Published:** 2026-05-04

**Authors:** Oman Nawabi, Nirav Pandya

**Affiliations:** 1 Union City, CA USA; 2 Department of Orthopaedic Surgery, University of California, San Francisco, CA USA

**Keywords:** Cricket, Injury, Prevention, Epidemiology, Mechanism, Management

## Abstract

**Purpose of Review:**

The objective of this review is to synthesize the current evidence that is available within the literature regarding injuries in cricket. This stems from epidemiology, mechanisms, management, prevention strategies, and the long-term outcomes of musculoskeletal injuries induced by cricket.

**Recent Findings:**

Prior literature has emphasized the importance of lumbar spinal health in fast bowlers with pathology onset. Yet, there is now an increased body of literature examining community cricket and adolescent players. Furthermore, injury prevention and workload monitoring for players, particularly female cohorts have also gained more recent attention as well.

**Summary:**

Synthesizing information from the literature has shown that fast bowlers tend to experience the highest injury rate. Most specifically, lumbar stress injuries and hamstring strains. Various other forms of injuries exist as well, such as to the shoulder and hand, and related fractures. There has traditionally been minimal research within the domain of female cohorts as well as adolescents. The biomechanics of cricket is gaining more awareness, and more studies are coming from sources outside of the UK and Australia.

## Introduction

Cricket is a globally played bat-and-ball sport at both the community and competitive levels, played between two teams in which one side bats to score runs while the opposing side bowls and fields to dismiss batters and limit scoring. The sport is played in multiple formats that differ in duration and workload demands. Test matches are played over several days, One-Day matches limit each team to 50 overs (sets of six deliveries) and the shorter Twenty20 (T20) format consists of 20 overs per side. Although the sport is primarily non-contact, injury rates remain high due to the aggressive nature of the sport. The act of throwing, running, catching, and hitting the ball can lead to the possibility of injury. Cricket is gaining more traction amongst female cohorts and is predominantly known as a male-dominated sport [1]. The sport is also played in various regions but is mostly centralized in South Asia [2]. As cricket continues to grow internationally, understanding the epidemiology, modes of injury, prevention, and management of cricket-related conditions is increasingly important for clinicians and sports medicine providers to improve the quality of life for players. The purpose of this review is to shed light on the injuries that occur in cricket to better understand the incidence rate of injuries, the mechanisms and causes, as well as general outcomes, both short and long-term.

## Epidemiology

The incidence of injury within the game of cricket is very broad, and the distinct modes of play will warrant different injury outcomes. For instance, at the highest level (T20), hamstring injuries are the most common, particularly amongst fast bowlers [3]. Furthermore, the location where the game is played can lead to certain injuries [3]. As noted in Orchard et al., playing in Australia had led to a greater incidence of hamstring injury compared to other locations [3]. Injury to the lumbar spine is well documented in numerous studies; however, the incidence rate is more common in younger bowlers [4]. Yet, seasonality has played a role as well with summer having a larger injury incidence [4]. Gender has also shown a distinct injury pattern. In one surveillance study in Australia, it was shown that over 7 seasons, males had a higher incidence rate than that of females [5]. Male players also had a greater number of match time-loss injuries [5]. Yet, with the game getting more attention amongst female players, the incidence of injury reporting amongst this demographic may be lower than what is occurring due to disparities regarding both medical care and training that warrant further examination.

In recreational cricket, the game is generally not played within a controlled environment in comparison to the elite level. In the non-organized space, injuries are not surveyed consistently, as was noted in a systematic review by McLeod et al. [6]. What has been reported is that there is a greater onset of fractures sustained amongst players, and most cases treated in a United States emergency department were injuries caused by the ball [7]. These may lead to the question of the safety protocols and potential coaching methods at the community level. This is particularly important as concussions and head injuries have also been reported within the literature, mostly presenting as contusions [8]. Upper extremity injuries are frequently observed as well, particularly among fielders, with injuries involving the hands and fingers [9]. Several studies have indicated that roughly one-third of all injuries in recreational cricket affect the upper extremities, which is significant as they account for a time loss from play [9]. These findings collectively demonstrate injury patterns at this level, as well as variation based on player position and level of organization. Table 1 summarizes injury incidence by population and level of play.

**Table 1:** Epidemiology of cricket injuries

Author (Year)	Population	Study Type	Significant indications
Orchard et al. 2016	Elite Male Cricketers	Surveillance over 10 seasons	Injury rate: 155 per 1,000 days of play. Annual injury rate: 64 per 100 players/season. Hamstring strain is most common at 8.7 per 100 players/season.
Hill et al. 2019	Elite Male and Female Cricketers	Epidemiological study, Cohort Size: 172 males, 106 females 2015–2016 season, 179 males, 98 females 2016–2017 season	Male matches: 92 head impacts, 29 concussions. Female matches: 15 head impacts, 8 concussions. Incidence per 1000 days: males 7.2 impacts/2.3 concussions; females 3.7 impacts/2 concussions.
Always et al. 2019	English Fast Bowlers	Epidemiological study, Cohort size: 368 2010–2016 seasons.	57 lumbar stress fractures; mean age of 22.8. Incidence: 0.16 per 10,000 deliveries; 2.46 per 100 fast bowlers/year. Workload of more than 37 + deliveries weekly, increased fracture risk 11 fold.
Keylock et al. 2022	Adolescent Fast Bowlers	Cross Sectional, Cohort Size = 40	Lumbar stress injury prevalence 20.5%. Injured bowlers showed delayed maturation (33% vs. 13% uninjured). Spinal development associated with injury risk.
Jacobs et al. 2024	Sub-Elite Female Cricketers	Prospective Cohort, Cohort size: 44 players, three teams.	Injury rate: 85.23 per 1,000 match days. Fast bowlers were most affected. Fielding caused 46.7% of injuries. Slightly more injuries during training than matches.
Forrester et al. 2021	Community Cricketers	Surveillance, Cohort size 485 injuries, with estimate of 13,729 injures, 2000–2019	Most common injuries: lacerations (24%), strains/sprains (21.4%), fractures (19.6%), contusions (13.2%). Upper extremity injuries (43.9%); head/neck (29.9%)
Dhillon et al. 2012	Professional Cricketers	Prospective study, Cohort size = 95, 2008–2009	16 upper extremity injuries; 10 occurred during fielding. Injuries: hands (11), elbow (3), shoulder (2). Middle phalanx fractures caused greatest time loss.

## Mechanisms & Pathophysiology

Even though cricket is primarily a non-contact sport, it still results in many types of injuries through unique mechanisms. For example, lumbar spine injuries are primarily evident amongst fast bowlers and begin at a very young age [10]. The continuous hyperextension of the lower back and workload can induce lumbar spondylolisthesis, and as a bowler ages, the changes can lead to worse spinal outcomes. It is important to note that biomechanics and spinal kinematics are becoming more important in understanding the onset of pathology amongst cricket players, particularly the fast bowlers [11]. Generating awareness can potentially lead to reduced injury rates if players were to adapt their approach in how they play. Players in the fielding position are generally susceptible to injuries of the hands and quadriceps, where the hand’s most prominent mechanisms are bruising or hematomas [12]. For the quadriceps, it is generally due to eccentric contraction of greater force [12]. These indications illustrate that upper and lower extremity injuries are present amongst the fielders and bowlers, despite the bowlers over-exhausting their upper limbs far more often than the general fielder, as they pitch continuously. There continues to be numerous studies within the literature indicating that the bowler position has the highest risk.

The mechanical nature of overhead throwing in the game of cricket between the stationary position and run-up position can further exacerbate risk potential [13]. In one study, there was an illustration in which the passive shoulder rotational range of motion was measured. The indications were that GIRD of over 20 degrees with lower shoulder force, combined with elbow compression found at cocking from a stationary position, had illustrated rotator cuff pathology [13]. A run-up approach in fielding illustrates an increase of hip flexion between 0 and 34% and 57–100% during one throw and lumbo-pelvic flexion between 57 and 65% [13]. Essentially, the mechanistic modality of throwing in the fielding position shows that the forces placed onto the fielder in the run-up position are doubled compared to if the ball were thrown in a stationary position at maximum external rotation [13]. The notion of throwing in the fielder or bowling position, even with good mechanics, the shoulder is still susceptible to labral tears and SLAP lesions [14]. The elbow is mentioned less often within the literature, as batters and bowlers can have lateral epicondylitis if equipment or the bat puts excessive strain on the elbow [14].

Although the upper and lower extremities are most prone to injury in the game of cricket, head injuries have a prevalence in the sport. Some positions are more susceptible to injury of the head than others, most specifically the batter position [15]. In one epidemiology study, the most common mechanism of injury was the ball impacting the helmet of the batter by a fast bowler, and uniquely, this was not shown to be the case amongst female cricket players [15]. It can be noted that slower bowling is more prevalent amongst women cricketers, as this variable can lead to a different result in contrast to men’s cricket [15]. Outside of concussions, traumatic injuries are also present on the head and neck of players [8]. The pathology is induced like concussions with the ball hitting a player, or a fielder diving and hitting their head on the ground [8]. Table 2 indicates the collective data from various studies showing the mechanism of injury and other key findings.

**Table 2:** Mechanisms and pathophysiology of cricket injuries

Author (Year)	Population	Injury	Mechanism	Significant Findings
Keylock et al. 2022	Adolescent Fast Bowlers	Lumbar stress injury/fracture	Hyperextension of the trunk combined with high ground reaction forces.	Difference in hip internal rotation of the contralateral leg can alter throwing kinematics, inducing greater strain. Workload intensity is a significant variable, as overuse can cause lumbar strain.
Alway et al. 2019	English fast bowlers	Lumbar spine	Extensive workload on the lumbar spine and increased seasonal frequency.	Lack of vertebral maturity is correlated with higher fracture susceptibility.
Orchard et al. 2017	Professional Australian Cricketers	Hamstring strain	Sprinting and overload of the hamstring	Season timing is correlated with fracture onset. Overuse of the lumbar spine inevitably leads to pathological onset. Speed and longevity of play contribute to injury risk. Run-ups increase hamstring onset, and increased sprinting speed leads to greater load on the lower extremities.
Jacobs et al. 2024	Sub-Elite Female Cricketers	Multiple body parts	Shoulder pathology occurs due to distraction forces. Increased left lateral flexion relative to the pelvis is associated with back pain. Hand pathology is induced by diving and catching.	Findings show a broad spectrum of injuries among female cricketers. Injury modes are consistent with trends in the literature. Different techniques may modify injury risk factors.
Eunson et al. 2020	Elite Australian Cricket Players	Head & Neck	Direct impact by the ball or head injury sustained by hitting the ground during fielding.	Injury rates are lower in sub-elite female players than elite players. Trends remain inconsistent due to limited data on helmet effectiveness in traumatic head injuries. The neck often remains unprotected during matches, supporting the need for improved protection.
Pardiwala et al. 2018	Cricket players from England, South Africa, Australia, the West Indies and India	Hand	Catching and diving induce hand pathology. External digits are most susceptible to fracture. Mechanisms of injury are not well described in studies.	Bruising and hematoma are the most common nonspecific hand injuries. Distal interphalangeal joint dislocation is the most common specific injury.

## Management & Long-Term Outcomes

Naturally, as a byproduct of playing competitive sports, injuries are inevitable. Understanding how to delegate treatment and ensuring adherence to said treatment will ensure a greater rate of return to play. For some, however, the return to play can be even longer for pathologies related to the spine. For instance, it can take between 7 and 11 months to return to professional cricket after lumbar surgery due to stress fractures from fast bowling [16]. However, most of these fast bowlers tend to return to their former performance before the onset of pathology [16]. The degree of management of lumbar conditions is uniquely complex for elite players, as they would need to avoid playing time for improvement. The use of bracing or other conservative measures has not shown a great change in outcomes unless the conditions were exaggerated [17]. A consistent reduction in play time can be a net negative for a cricket player.

Regarding shoulder injuries, the transition to a higher intensity modality of cricket leads to a change in how aggressive players are [14]. This can skew what can be deemed more commonplace in the community or at a less competitive level. For example, it is known that shoulder injuries will have a higher frequency of onset as more professional cricket players play in a T20 format [18]. Fielders typically experience a great deal of shoulder problems due to the nature of throwing the ball or diving peculiarly. Being aware of changes to the game and injury incidence can lead to greater methods of pathology management and assessing for long-term outcomes. The return to play for shoulder conditions is very broad, and most are caused by the fielding position [19]. In one study of female cricketers, about 80% of the injuries are typically non-time loss injuries, and it is well known and established that recurrent injuries have the longest recovery time for return to play [19]. Long-term analysis appears to be less abundant across studies. Shoulder management is assessed by resting and anti-inflammatory intervention as well as steroid injection [14].

Regarding the knee, as it is usually not discussed in isolation across studies, it is managed through physiotherapy with steroid injections if needed, and in rare cases, through surgical intervention [14]. A cross-sectional study was performed to identify long-term conditions, such as osteoarthritis, in the population of elite players in comparison to the general population. Within this study, former elite cricketers were recruited through the Professional Cricketers’ Association and completed a self-reported questionnaire. The English Longitudinal Study of Ageing was used as the comparison group for the former cricketers in which members of the study had been diagnosed by a physician for their diseases. The resulting indications demonstrated that there is a higher prevalence of osteoarthritis of the knee, hip, and hand, as well as increased rates of joint arthroplasty [20]. Despite the increase in osteoarthritis and anxiety, the study showed that there was no regret in participating in cricket and there were reports of lower rates of cardiovascular disease [20]. This finding suggests long-term satisfaction with cricket participation will persist despite the injuries sustained and the impacts they have had.

Within the study of Brooks et al., it is mentioned that hand injuries are one of the most prominent within the game of cricket [21]. Knowing that players are prone to hand fractures through the fielding position at a high rate, as collected through numerous studies, preventative measures are of utmost importance [21]. Most of the fractures occur on the outside of the hands, and the informed frequency of injury will allow clinicians to predict return to play rates and whether surgical indications are necessary [21]. The use of gloves is present within cricket, but the rate of hand injury seems to contrast with how useful they may be. The return to unrestricted play is similar between conservative treatment and surgical treatment [21]. Methods of clinical management of hand fractures or injuries can be splinting, open reduction internal fixation, or trans fixation of joints with a Kirschner wire [14]. General treatment for symptoms of the hand is physiotherapy [14]. There appears to be less data within the literature covering a broader scope of return to play times for cohorts. Table 3 indicates the methodology of approach regarding the management of injuries and what is to come long-term.

**Table 3:** Management and long-term outcomes of cricket injuries

Author (Year)	Population	Injury	Management	Long-Term indications/Findings
Jones et al. 2018	Former Elite Cricketers	Hip & Knee	Total joint replacement of the knee or hip	Retired cricketers demonstrated higher rates of arthroplasty indications, with hip involvement in 14.7% and knee involvement in 10.7% compared with the general population. A 51.3% higher prevalence of arthritis was also observed.
Pritchard et al. 2025	Elite Female Cricketers	Shoulder	Reduce throwing volume with modification.	Players were able to continue participation in batting or bowling despite shoulder pathology. Shoulder instability and glenohumeral injury accounted for most of the pathology, often requiring approximately two weeks of rest, while surgical cases required approximately 13 months for return to play.
Schouten et al. 2018	Professional Male Cricketers	Lumbar Spine	Lumbar stress fracture patients had undergone 2–6 months of rest and rehabilitation. Failed conservative measures warranted surgical indication of spondylolysis.	The cohort had an average age of 26 years, with return to play occurring approximately eight months post-surgery. Pars defects were present in 32% of fast bowlers, exceeding rates in the general population. Post-surgical outcomes were generally favorable, though further research is needed.
Shafi et al. 2014	Elite Cricket players	Multiple body parts	Indications of physiotherapy, steroid intervention, surgical indications and splinting.	Repetitive throwing contributes to rotator cuff pathology, including supraspinatus injury. Strengthening of external rotators and eccentric training recommended. Elbow injuries typically resolve with six weeks of physiotherapy, while hand injuries may require three to twelve weeks depending on severity. Rehabilitation programs are critical for reducing future injury risk.

## Prevention

Prevention is becoming increasingly more apparent within cricket literature. As management has continued to improve, it is now the case that the call for prevention is increasing. A cross-sectional study in Nepal had found that male cricketers had a much higher knowledge base than female cricketers, and at the elite level, there was an even greater knowledge base of preventative measures [22]. Given the increase in participation of female cricketers, these findings suggest that injury prevention programs should have more prioritization for female players to reduce this knowledge gap. This study reinforces the prior indication that community-level cricket is more susceptible to injury incidence due to a less controlled environment and a lack of potential knowledge on how to prevent injury. The study had also shown that the availability of medical staff on site positively correlated with a higher base of knowledge [22]. A brief analysis on the use of protective gear showed that kinematic forces and acceleration were measured to elaborate on the efficacy of the cricket helmet [23]. The findings indicate that up to 60% of the total pressure is reduced with the use of the helmet and can reduce the onset of concussions for players [23]. This reinforces the need for adherence to safety equipment.

In a randomized controlled trial amongst community adolescent cricketers, an exercise-induced injury prevention program was analyzed, and the results showed that risk factors were modified [24]. Outside the use of equipment, workload is a variable that can aid in reducing injury onset to players. Adolescent fast bowlers with a mean age of 14.7 were assessed for injury rates in a study by Dennis et al., dependent upon bowling load [25]. A quarter of the bowlers reported overuse, and it was shown that bowlers who threw more frequently had a higher rate of injury [25]. Less rest in between bowling increased the likelihood of injury [25]. This trend appears to be consistent within the literature, as game type and population can lead to a greater onset of injury. The more aggressive the game, the higher the injury onset. Younger bowlers may need to tread more carefully when bowling, as prior studies indicated that spine maturation was a factor for a higher rate of injury. Insight into biomechanics should be of greater importance for this respective population, as these studies continue to highlight this.

## Conclusion

Cricket contains a variety of injuries that are spread out through different player positions. The evidence of safety and preventative measures with medical access is greater at the elite level. Trends within the literature across years of study have tended to be consistent regarding pathology onset, particularly the lumbar spine. Female and adolescent cohorts are also underrepresented, with only studies covering them more recently. Further longitudinal studies amongst female cohorts are recommended. Long-term outcomes have been illustrated in some cases, such as osteoarthritis. However, awareness of life after injury from cricket or long-term cricket playing should be investigated further. Further study is also needed regarding biomechanics and injury prevention programs to improve short, mid and long-term quality of play. The awareness of injuries and their anticipation is paramount to ensure safer play at every level. This will aid in preventing injuries that can be very drastic and potentially alter the quality of a player’s life, inside and outside the game of cricket.

## Key References

- Jacobs J, Olivier B, Brandt C. Injury profiles in sub-elite Women’s Cricket: Exploring incidence, prevalence, nature, onset and body region. Phys Ther Sport. 2024 May; 67:125–130. doi: 10.1016/j.ptsp.2024.04.006. Epub 2024 Apr 23. PMID: 38701662.
  - ○ Prospective cohort study that observed outcomes longitudinally for women cricketers and reported injuries as they occurred.
- Sharma, P., Keshav, K., Mishra, P. et al. Epidemiological study of injury profile in cricketers of South-Asian Countries: a systematic review and meta-analysis. Discov Public Health 22, 252 (2025). 10.1186/s12982-025-00642-2.
  - ○ Systematic review and meta-analysis that highlights injuries amongst South-Asian cricketers.
- Eunson TH, Saw AE, Kountouris A, Orchard JW. Traumatic Head and Neck Injuries in Elite Australian Cricket Players: Retrospective Analysis from 12 Seasons. Indian J Orthop. 2023 Jun 6;57(10):1584–1591. doi: 10.1007/s43465-023-00916-4. PMID: 37766950; PMCID: PMC10519901. 
  - ○ Retrospective cohort study that reviewed 12 seasons of reported head and neck injuries for Australian male and female players.
- Keylock L, Alway P, Felton P, McCaig S, Brooke-Wavell K, King M, Peirce N. Lumbar bone stress injuries and risk factors in adolescent cricket fast bowlers. J Sports Sci. 2022 Jun;40(12):1336–1342. doi: 10.1080/02640414.2022.2080161. Epub 2022 May 28. PMID: 35635278. 
  - ○ Prospective cohort study that monitored adolescent fast bowlers during a season and recorded injuries as they occurred.
- Shafi M. Cricket injuries: an orthopaedist’s perspective. Orthop Surg. 2014 May;6(2):90 − 4. doi: 10.1111/os12104. PMID: 24890289; PMCID: PMC6583247. 
  - ○ Review that emphasizes injuries amongst cricketers that are assessed by an orthopedist.
- Orchard JW, Saw R, Kountouris A, Redrup D, Farhart P, Sims K. Management of lumbar bone stress injury in cricket fast bowlers and other athletes. S Afr J Sports Med. 2023 Jun 5;35(1):v35i1a15172. doi: 10.17159/2078-516X/2023/v35i1a15172. PMID: 38249766; PMCID: PMC10798616.
  - ○ Review of injuries focused on the lumbar spine such as pars defect and stress reaction.
- Mansingh A. Shoulder Injuries in Modern Cricket: Should an Increase Be Anticipated? Indian J Orthop. 2023 Aug 23;57(10):1561–1564. doi: 10.1007/s43465-023-00963-x. PMID: 37766960; PMCID: PMC10519877. 
  - ○ Review of injuries to the shoulder that investigates the change in intensity of modern cricket as a causal variable.
- Pritchard G, Deshmukh P, Saw AE, Beerworth K, Sims K. Shoulder injuries in elite female cricket players: Insights from eight seasons. Shoulder Elbow. 2025 May 22:17585732251344257. Doi: 10.1177/17585732251344257. Epub ahead of print. PMID: 40417405; PMCID: PMC12098314. 
  - ○ Retrospective cohort study of female cricketers over the course of eight seasons, investigating shoulder injuries and their causation.
- Pandey M, Bhusal R, Acharya A, Sharma S, Bhattarai N, Acharya A, Jamarkattel S, Joshi A. Assessment of sports injury knowledge and preventive practices among national-level professional cricket players in Nepal: a cross-sectional study. BMC Sports Sci Med Rehabil. 2025 Jul 1;17(1):154. Doi: 10.1186/s13102-025-01203-5. PMID: 40597316; PMCID: PMC12220397. 
  - ○ Cross-sectional study measuring the knowledge base of male and female cricketers in Nepal about injury and prevention.

## Data Availability

No datasets were generated or analysed during the current study.
